# DelPhi: a comprehensive suite for DelPhi software and associated resources

**DOI:** 10.1186/2046-1682-5-9

**Published:** 2012-05-14

**Authors:** Lin Li, Chuan Li, Subhra Sarkar, Jie Zhang, Shawn Witham, Zhe Zhang, Lin Wang, Nicholas Smith, Marharyta Petukh, Emil Alexov

**Affiliations:** 1 Physics Department, Computational Biophysics and Bioinformatics, Clemson University, Clemson, SC, 29642, USA; 2 Department of Computer Science, Clemson University, Clemson, SC, 29642, USA

**Keywords:** DelPhi, Poisson-Boltzmann equation, Implicit solvation model, Electrostatics, Biological macromolecules, Software

## Abstract

**Background:**

Accurate modeling of electrostatic potential and corresponding energies becomes increasingly important for understanding properties of biological macromolecules and their complexes. However, this is not an easy task due to the irregular shape of biological entities and the presence of water and mobile ions.

**Results:**

Here we report a comprehensive suite for the well-known Poisson-Boltzmann solver, DelPhi, enriched with additional features to facilitate DelPhi usage. The suite allows for easy download of both DelPhi executable files and source code along with a makefile for local installations. The users can obtain the DelPhi manual and parameter files required for the corresponding investigation. Non-experienced researchers can download examples containing all necessary data to carry out DelPhi runs on a set of selected examples illustrating various DelPhi features and demonstrating DelPhi’s accuracy against analytical solutions.

**Conclusions:**

DelPhi suite offers not only the DelPhi executable and sources files, examples and parameter files, but also provides links to third party developed resources either utilizing DelPhi or providing plugins for DelPhi. In addition, the users and developers are offered a forum to share ideas, resolve issues, report bugs and seek help with respect to the DelPhi package. The resource is available free of charge for academic users from URL: http://compbio.clemson.edu/DelPhi.php.

## Background

Electrostatic interactions play an important role in biological systems [1-5] because biomolecules are composed of atoms carrying partial charges. Since the typical distances between atoms inside a biomolecule are on the order of several angstroms, the resulting electrostatic energy could be very large and be the major component of total energy [6-8]. Even more, at large distances, the electrostatic energy is the dominant component of the energy because all other components vanish. Since electrostatic interactions are the dominant factors for both inner- and inter- molecular interactions, accurate calculations of electrostatic potential and energies are crucial to reveal the mechanisms of many different biological phenomena, such as protein folding [9], protein-protein and protein-DNA binding [10-13], pKa shifts in proteins [14-17] and RNAs [18], and many others [19,20].

Biomolecules function in water, which makes the calculation of electrostatic potential a challenge due to the complexity of the water environment [21,22]. Various models have been developed to calculate electrostatic energy of biomolecules in the presence of surrounding water. These models can be categorized into two types [23-28]: explicit [29,30] and implicit [31-34] solvation models. Explicit solvation models treat the solvent as individual water molecules and are believed to be more accurate but time-consuming, and therefore, are usually suitable for systems involving small to medium size biomolecules. However, most biological systems are large and contain a huge amount of water molecules. To calculate electrostatics in such large systems, more computationally efficient algorithms are typically applied. These methods, called implicit solvation methods, treat the water phase as a continuum medium. The Poisson-Boltzmann Equation (PBE) is one of the most successful implicit solvation models and was implemented in many well-known programs, such as DelPhi [35,36], APBS [37,38], MEAD [39], ZAP [40], PBEQ [41], MIBPB [42], UHBD [43], ITPACT [44], and several others. Among the above mentioned software, DelPhi has been proven to be among the best performers due to its unique features, such as capabilities of handling systems with multiple dielectric constants, modeling systems with multivalent ions, rapidly constructing molecular surfaces, calculating charged geometric object systems, and deriving ion concentration and dielectric maps.

In addition to the DelPhi source code and executable files which are free for academic users, several other resources are provided [45]. DelPhi’s distribution is adapted for different operating systems and provides a series of examples along with the user manual. Parameter files for four of the most widely used force fields [46-51] are also provided on the DelPhi website, which gives the users more options to explore various scenarios and to port snap-shots from molecular dynamics simulations into DelPhi calculations. The DelPhi forum [52] is set up for users to exchange ideas, discuss and solve problems, post suggestions for further development, and other DelPhi related issues. Furthermore, the DelPhi web server is also developed [53], allowing non-experienced users to quickly perform calculations on their selected structures. The DelPhi suite offers various tools and plugins [54-56] developed by other researches which utilize DelPhi to address biological questions as well.

### Implementation

The PBE model treats solvent as a continuum medium with high dielectric constant. Biomolecules are considered as low dielectric cavities made of charged atoms. Ions in the water phase are modeled as non-interacting point charges and their distribution obeys the Boltzmann law. Utilizing the Gauss-Seidel method, DelPhi solves both linear and nonlinear PBE in a cube of N × N × N grid points [57,58].

**Figure 1:**
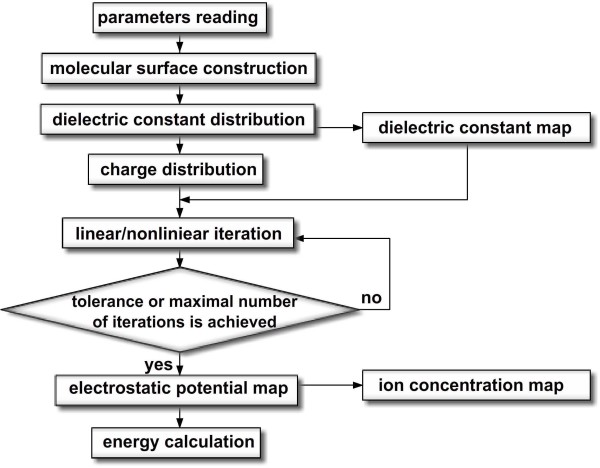
Flowchart of the DelPhi program.

The overall architecture of DelPhi is shown in the flowchart in Figure 1. The core of DelPhi are the subroutines described below. The DelPhi specific input parameters are provided in a parameter file and the input data is read from three input files: coordinates, charges and radii files. First of all, user-desired parameters are specified in a parameter file. This parameter file controls the initial set up of the run and provides the file names of the coordinate, charge and size files. One can specify the scale and filling percentage. DelPhi automatically calculates the necessary grid size, given the scale and filling percentage. According to the coordinate and size files, DelPhi generates the molecular surface by utilizing a rapid construction method. Dielectric constant values, which form a three dimensional dielectric constant map, are given at grid midpoints. Next, the charges are assigned to atoms and then distributed onto the grid points. Using distributed charges and the dielectric constant map, DelPhi initiates the iterations to solve the linear or nonlinear PBE. Iterations stop when the user specified tolerance or the maximal number of iterations is achieved. DelPhi then produces the three dimensional electrostatic potential map. Using this potential map, DelPhi can also generate the ion concentration map. If requested, the dielectric constant, electrostatic potential, and ion concentration maps can be saved into files and rendered by visualization software. Using the charge distribution and potential map, the Coulombic, grid and solvation energies are calculated.

In addition to the routines described above, DelPhi also has some unique functionalities, such as handling multiple dielectric constants and mixed multivalent ions, rapid constructing molecular surface, generating geometric objects and performing calculations. The multiple dielectric constant method divides the bio-molecular system into different parts, and assigns each part a specific dielectric constant allowing the difference in conformational flexibility to be modeled by different dielectric constants as illustrated in Ref. [59]. DelPhi can also model solvents with mixed ions, which may have different concentrations and valences. In order to speed up the process of generating a molecular surface, a rapid surface construction method has been developed in DelPhi, which implements the marching cube algorithm to construct the surface quickly and accurately. These features are described in detail in [35]. Four types of basic objects are now available in DelPhi package: sphere, cylinder, cone and box. Using the object functions, together with the multiple dielectric constants option, users can create complex geometric structures with different dielectric constants and shapes [35]. The DelPhi package is written in the FORTRAN and C language and can be compiled as single or double precision according to the practical usage. DelPhi version 5.1 is now available on different operating systems, including Windows, Linux and Mac. Although many useful functions and options have been implemented, DelPhi is still user friendly and compatible. Several other groups have developed third-party plugins and tools to utilize DelPhi on other software, such as UCSF Chimera [56], DelEnsembleElec (GUI and a plugin for VMD [55]), Biskit [54], and others [60].

## Results and discussion

There are several important characteristics used to classify methods and software packages: accuracy, rate of convergence and speed of calculations. In the next several subsections, we performed several tests on DelPhi with respect to these features.

### Accuracy test

Accuracy is one major concern of any numerical solver. In order to measure the solver’s accuracy and demonstrate that it solves the exact problem, the solver is usually tested on simple examples for which analytical solutions exist. In this subsection, three simple examples with regular geometry were selected. We compared their analytical solutions with the numerical ones obtained by DelPhi. Since the computational algorithm does not distinguish simple geometrical objects from real biological macromolecules with more complex shape, it indicates that DelPhi solves the PB equation and produces close numerical approximations to the real solutions. The following constants were fixed in all examples: elementary charge $e=1.602176565\times 10^{-19}C$_,_ vacuum permittivity $\varepsilon_{0}=8.8541878176\times 10^{-12}F/m,$ Boltzmann constant $k=1.38\times 10^{-23}J/K$ and temperature T=297.33K_._

### *A sphere in water*

**Figure 2:**
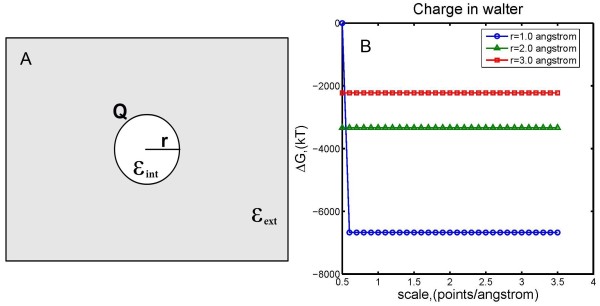
**(A) Schematic illustration of example 1: A charged sphere with low dielectric constant is inside a media with a high dielectric constant.** (**B**) Electrostatic component of the solvation energies calculated by DelPhi against the analytical solution.

The first example presents a charged atom with a lower dielectric constant *ϵ*_int_ immersed in a continuum media with a higher dielectric constant *ϵ*_*ext*_. In this example, the electrostatic component of solvation energy Δ*G*^*sol*^ can be obtained by the Born formula and is explicitly given by

(1) $$\Delta G^{\mathrm{sol}}=-\frac{Q^{2}}{2\cdot 4\cdot \pi \cdot \varepsilon_{0}}\cdot \frac{1}{r}\left(\frac{1}{\varepsilon_{\mathrm{int}}}-\frac{1}{\varepsilon_{\mathrm{ext}}}\right)\text{,}$$

where *Q* and *r* are charge and radius of the charged atom, as shown in Figure 2A.

Setting *ε*_*int*_ = 4.0, *ε*_*ext*_ = 80.0 and *Q* = 10.*e*, values of Δ*G*^*sol*^ obtained by Equation (1) are −6673.71kT, -3336.86kT and −2224.57kT (rounded to two decimals) for radii *r* = 1 Å, 2 Å and 3 Å, respectively. These values were compared to those obtained by DelPhi at various scales (points/Å) and the results are shown in Figure 2B. It is clear that no visual difference can be observed when the scale is greater than 0.5 points/Å.

### Two charges in a protein

**Figure 3:**
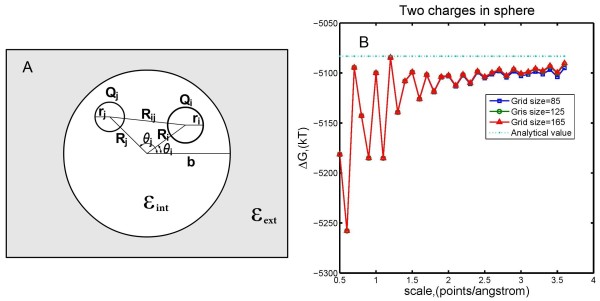
**(A) Schematic illustration of example 2: a cavity with low dielectric constant is inside a media with high dielectric constant.** Two charged atoms are located inside the cavity. (**B**) Electrostatic component of solvation energy obtained from DelPhi compared with analytical solution.

The next example, shown in Figure 3A, describes a spherical protein with radius *b* locating inside a media without any ions. Dielectric constants in the interior and exterior of the protein are denoted by *ε*_int_ and *ε*_*ext*_ again. Two atoms with radii *r*_*i*_ and *r*_*j*_ are centered at points with polar coordinates $\left(R_{i},\theta_{i}\right)$ and $\left(R_{j},\theta_{j}\right)$. These two atoms are placed inside the protein and are assigned charges *Q*_*i*_ and *Q*_*j*_, respectively. This example has been studied by Barry Honig and co-workers [1]. The analytical solution of the electrostatic component of solvation energy Δ*G* is composed of four terms:

(2) $$\Delta G=\Delta G_{\mathrm{ij}}^{c}+\Delta G_{\mathrm{ij}}^{\mathrm{pol}}+\Delta G_{\mathrm{ii}}^{\mathrm{self}}+\Delta G_{\mathrm{jj}}^{\mathrm{self}}\text{,}$$

where $\Delta G_{\mathrm{ij}}^{c}$ is the Coulombic energy of atom *ij*$\Delta G_{\mathrm{ij}}^{\mathrm{pol}}$ is the pairwise polarization interaction energy, $\Delta G_{\mathrm{ii}}^{\mathrm{self}}$ is the total self-energy of atom *i* and $\Delta G_{\mathrm{jj}}^{\mathrm{self}}$ is the total self-energy of atom *j*. Energies on the right-hand side of Equation (2) can be calculated by

(3) $$\begin{array}{c} \Delta G_{\mathrm{ij}}^{c}=\frac{1}{4\cdot \pi \cdot \varepsilon_{0}}\cdot \frac{Q_{i}\cdot Q_{j}}{\varepsilon_{\mathrm{int}}\cdot R_{\mathrm{ij}}}\text{,}\;\\ \Delta G_{\mathrm{ij}}^{\mathrm{pol}}=\frac{1}{4\cdot \pi \cdot \varepsilon_{0}}\cdot \frac{Q_{i}\cdot Q_{j}}{\varepsilon_{\mathrm{int}}}\cdot \sum_{n=0}^{\infty }B_{n,ij}\cdot P_{n}\left(\cos\left(\theta_{i}-\theta_{j}\right)\right)\\ \Delta G_{\mathrm{ii}}^{\mathrm{self}}=\frac{1}{4\cdot \pi \cdot \varepsilon_{0}}\cdot \frac{Q_{i}{}^{2}}{2\cdot \varepsilon_{\mathrm{int}}}\cdot \sum_{n=0}^{\infty }B_{n,ii}\text{,} \end{array}\text{,}$$

where $B_{n,ij}=\frac{{\left(R_{i}\cdot R_{j}\right)}^{n}}{b^{2\cdot n+1}}\cdot \frac{\left(n+1\right)\cdot \left(\varepsilon_{\mathrm{int}}-\varepsilon_{\mathrm{ext}}\right)}{\left(n+1\right)\cdot \varepsilon_{\mathrm{ext}}+n\cdot \varepsilon_{\mathrm{int}}}$ and $P_{n}\left(\cos\left(\theta_{i}-\theta_{j}\right)\right)$ is the nth order Legendre polynomial.

Substituting $Q_{i}=Q_{j}=10\cdot e$, $R_{i}=R_{j}=5\sqrt{2}$ Å,$\theta_{i}=\pi /4$, $\theta_{j}=3\pi /4$, b=10 Å, r=1 Å, $\varepsilon_{\mathrm{int}}=2.0$ and $\varepsilon_{\mathrm{ext}}=80.0$ into Equations (2) – (3) yields ΔG=−5083.19 kT after rounding to two decimals. Numerical calculations were performed at grid size = 85, 125, and 165 and various scales. The numerical results, together with the value of Δ*G*, were compared and shown in Figure 3B. One can see that the numerical solutions converge to the real solution quickly as scale increases for all three tested grid sizes.

### A sphere in semi-infinite dielectric region

**Figure 4:**
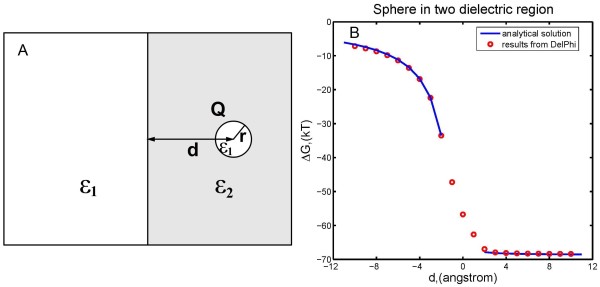
**(A) Schematic illustration of example3: the space is divided by media with two different dielectric constants.** A sphere with low dielectric constant *ε*_1_ is initially positioned in region with high dielectric constant *ε*_2_ and moves into the region with low dielectric constant *ε*_1_. (**B**) Electrostatic component of solvation energy derived from DelPhi compared with analytical solutions. Blue solid lines represent analytical solutions, red circles represent numerical results from DelPhi.

The third example considers a space split into two regions with different dielectric constants, as shown in Figure 4A. Dielectric constant in the left region is *ϵ*_1_ and that in the right region is *ε*_2_ ($\varepsilon_{2}>\varepsilon_{1}$). A sphere with radius *r* and dielectric constant *ϵ*_1_ is initially positioned in the right region. The distance between the center of the sphere and the boundary of two regions is denoted by *d*. Let the sphere move towards the boundary and eventually get into the left region. We consider *d* > 0 when the center of the sphere is still in the right region and *d* < 0 when it is in the left region. The sign of *d* indicates the position of the sphere. During the moving process of the sphere, except the moment when the sphere intersects both regions (i.e., |d|≤r), the electrostatic component of the solvation energy Δ*G* can be analytically expressed as a function of distance *d*

(4) $$\Delta G=\{\begin{array}{cc} \frac{1}{4\cdot \pi \cdot \varepsilon_{0}}\cdot \frac{Q^{2}}{2\cdot r}\cdot \left(\frac{1}{\varepsilon_{2}}-\frac{1}{\varepsilon_{1}}\right)+\frac{1}{4\cdot \pi \cdot \varepsilon_{0}}\cdot \frac{\varepsilon_{2}-\varepsilon_{1}}{\varepsilon_{2}+\varepsilon_{1}}\cdot \frac{Q^{2}}{4\cdot \varepsilon_{2}\cdot d}, & \text{when}\;d>r\text{,}\;\\ \frac{1}{4\cdot \pi \cdot \varepsilon_{0}}\cdot \frac{\varepsilon_{2}-\varepsilon_{1}}{\varepsilon_{1}+\varepsilon_{2}}\cdot \frac{Q^{2}}{4\cdot \varepsilon_{1}\cdot d}\text{, } & \text{when}\;d<-r\text{.} \end{array}$$

The blue curve in Figure 4B represents the function Δ*G*(*d*)(Equation 4), here we set $\varepsilon_{2}=80.0$, $\varepsilon_{1}=2.0$, r=2 Å, Q=1⋅e. Numerical results obtained by running DelPhi at a series of discrete *d* values are shown by red circles in Figure 4B and fit the curve very well. Our tests in this example indicate that DelPhi delivers accurate numerical approximations to the real solution.

### Rate of convergence

The rate of convergence is another major concern from the numerical point of view. DelPhi utilizes the Gauss-Seidel iteration method, along with the optimized Successive Over-Relaxation method [58], to solve PBE in a cube. The solution is more accurate when the cube is discretized into finer grids. In order to determine the minimal requirement of computational time cost for DelPhi to achieve results within a desired accuracy, a series of tests were designed and implemented on a typical protein of medium size, namely the bovine alpha-chymotrypsin-eglin C complex [PDB:1ACB], to demonstrate the performance of DelPhi.

**Figure 5:**
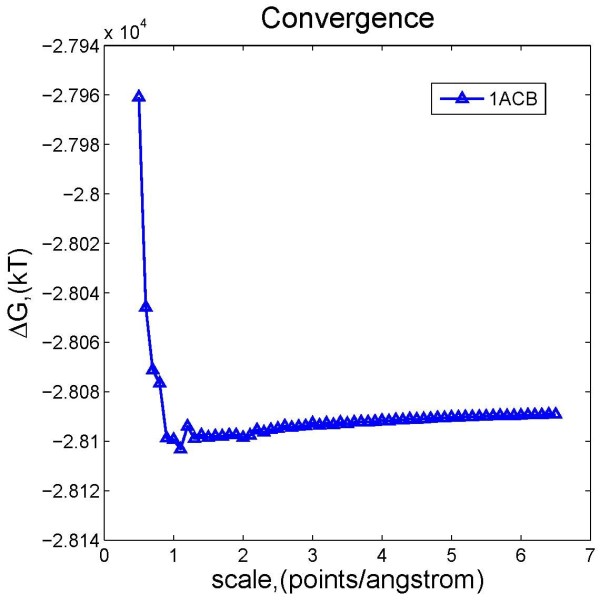
Electrostatic solvation energies of 1ACB obtained from DelPhi with respect to different scale values.

These tests were performed by varying the value of scale from 0.5 points/Å to 6.5 points/Å at step size 0.1 points/Å. Noticing that scale is the reciprocal of grid spacing, this means larger scale results in finer discretization of the cube. The filling percentage of the cube, *perfil* = 70%, was fixed in all tests. The resulting electrostatic component of solvation energy Δ*G* as a function of scale is shown in Figure 5.

The energy calculations on the structure of 1ACB show that the approximate scale threshold is 1 points/Å. At scale larger than the threshold, the calculated electrostatic component of solvation energy is almost scale-independent and reaches steady value of −28089 kT. Achieving such steady value at scale 1 – 2 points/Å demonstrates the robustness of the algorithm that calculates the electrostatic component of solvation energy, so termed the corrected reaction field energy method [35].

### Speed of calculations

DelPhi utilizes various algorithms and modules to calculate electrostatic potential and energy. The basic modules include generating molecular surface, calculating electrostatic potential distribution and obtaining the corresponding electrostatic energy. The speed of calculations for each of these modules depends on various factors, such as scale, number of atoms/charges, shape/net charge of the molecule. In order to reveal their impact on the performance of DelPhi from the users’ point of view, we first tested DelPhi on a particular protein complex with fixed filling of the cube and increasing scale, and next, tested DelPhi on multiple proteins with fixed scale. All calculations were performed on the same type of CPU, Intel Xeon E5410 (2.33 G Hz), on the Palmetto cluster [61] at Clemson University. Each run was repeated 5 times and the average is reported here in order to reduce unexpected fluctuations caused by system workload at run time. The resulting CPU time against scale and protein size, are reported as follows.

#### Speed of calculations as a function of scale

**Figure 6:**
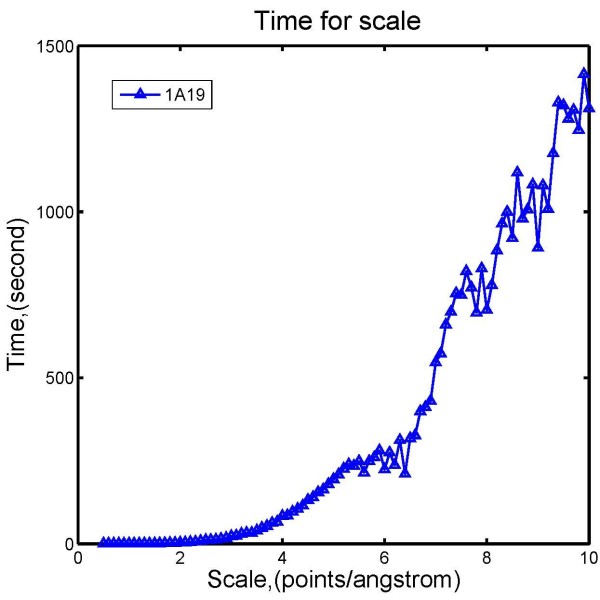
Electrostatic solvation energies calculation time of 1A19 with respect to different scale values.

In this case, we calculated the energy of *barstar* [PDB:1A19] using DelPhi, with scale values increasing from 0.5 to 10 points/Å at step size 0.1 points/Å. The *perfil* value was set to 70% regardless of the changing scales to keep the filling of the cube fixed. The resulting CPU time, plotted as a function of scale, is shown in Figure 6. It can be seen that the computational time rapidly increases with the scale, because the corresponding grid size increases as well. However, at scale of 2–4 points/Å. DelPhi is still very fast, resulting in runs of about a second to several seconds.

#### Speed of calculations as a function of protein size

**Figure 7:**
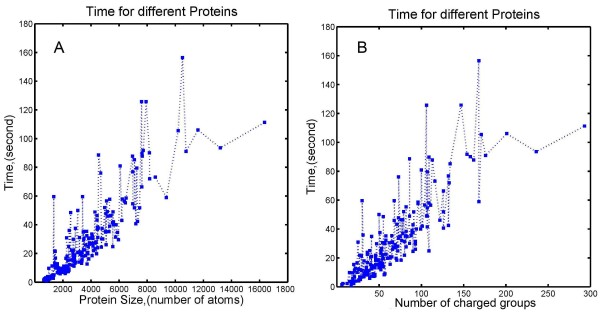
Electrostatic solvation energies calculation time of 200 proteins with respect to (A) number of atoms and (B) number of charged groups of each protein.

To evaluate the influence of protein size, 200 proteins from Zhang’s benchmark [62] were selected and tested using DelPhi. The number of atoms in these proteins range from 639 to 16361. For each protein, the scale was set to be 2 points/Å and *perfil* was 70%, which are reasonable and common values for calculations on real biomolecules. The resulting calculation time is plotted as a function of protein size in Figure 7A. One can see that the resulting computational time, in general, increases with the size of the protein. However, the energy of the largest protein in the dataset, composed of more than 16,000 atoms, was calculated in less than 120 s, almost the same time needed for small proteins made of about 8,000 atoms. This indicates that other factors, such as the number of charged groups, may play important roles as well. To test such a possibility, the number of charged residues for each protein was obtained and the calculation time is plotted against it (Figure 7B). The resulting plot is not much different from Figure 7A and the calculation time for the protein with the largest number of charged groups is not necessarily the longest one. This illustrates that the computation time is a complex function depending on the combination of protein size, number of charges, shape and many others.

Electrostatic energy calculations on large biomolecules usually cost more CPU time primarily due to two factors: Firstly, large biomolecules need a large cube and consequently more grids to be represented. Secondly, larger biomolecules contain more charged atoms and require more time to calculate the energy terms. However, the curve of the 200 proteins is not smooth, because there are several other factors which influence the calculation time. The size of the modeling cube depends not only on the atom number, but also on the molecule’s shape. A narrow and long molecule may need a larger cube than a spherical molecule even if their atom numbers are the same. The irregularity of molecular surface also affects the iteration time. A molecule with an irregular surface requires more iterations to converge than a molecule with a regular, smooth surface. Finally, a molecule with a higher charge needs more iterations than a molecule with a lower charge. Due to above reasons, larger molecules usually (but not necessarily) cost more time than smaller molecules to calculate the corresponding potential and energies.

### Effect of force field parameters (Charmm, Amber, OPLS and Parse)

**Figure 8:**
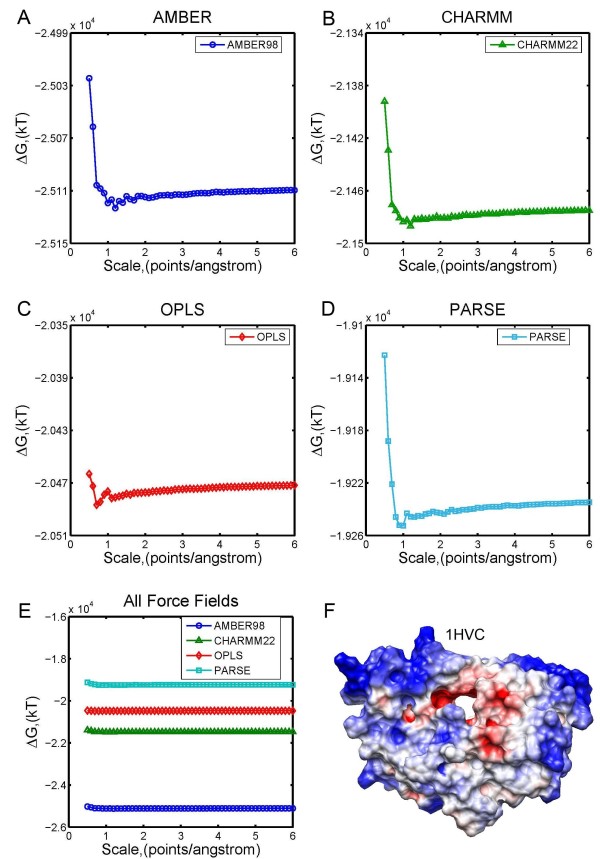
**Electrostatic solvation energy calculation of 1HVC using 4 different force fields.** (**A**)-(**D**) Calculated electrostatic solvation energies using AMBER, CHARMM, OPLS, and PARSE force fields. (**E**) All of the 4 results are shown in one figure to show the differences. (**F**) Electrostatic potential surface of HIV-1 protease [PDB:1HVC], generated by using AMBER force field.

It is well known that different force fields perform differently in protein folding [63-65]. It was also illustrated that the electrostatic component of binding free energy is very sensitive to force fields [66]. Because of that, it is desirable that DelPhi handles electrostatic calculations with different force fields on 3D structures obtained from the corresponding MD simulations. Currently, four widely used force fields are available in DelPhi package: AMBER98 [46], CHARMM22 [47], OPLS [48-50] and PARSE [51]. Here we calculated the electrostatic component of solvation energy of HIV-1 protease [PDB:1HVC] by using the above mentioned force fields (Figure 8). The *perfil* was set to be 70%, probe radius was 1.4 Å, the dielectric constants were set as 4.0 inside the protein and as 80.0 in the water, and the scales varied from 0.5 to 6.0 points/Å.

Results of the calculated electrostatic energies on 1HVC are shown in Figure 8, using AMBER, CHARMM, OPLS, and PARSE. The results reaffirm the previously made observations that calculations at very small scales are not accurate. However, once the scale is larger than 1 point/Å, DelPhi achieves convergence quickly and results are almost scale independent. There is a slight tendency that CHARMM and PARSE converge faster than other force fields, but the difference is small. At the same time, the electrostatic energies calculated using different force fields are quite different. When scale reaches 6 points/Å, the calculated energies are: -25109.58, -21474.99, -20471.78, -19234.74 kT for AMBER, CHARMM, OPLS, and PARSE, respectively. The largest difference in calculated energy is obtained by AMBER force field parameters versus others. Such a large difference should not be surprising since the force field parameters are developed with respect to the total energy, not just the electrostatic component. However, several studies [16,67,68] indicate that the energy difference remains even in the calculations of total energy, although the differences are smaller compared to the differences in the electrostatic component. The same is valid for calculations involving the difference of energies, as for example the electrostatic component of the binding energy [66]. It was shown [66] that the difference could be larger than 50 kcal/mol. These observations and the results presented in this work indicate the sensitivity of calculations with respect to the force field parameters and suggest that the outcome of the modeling should be tested with this regard.

## Conclusions

In this work, we described the DelPhi package and associated resources. DelPhi is a comprehensive suite including DelPhi website, web server, forum, DelPhi software and other tools. Several tests were performed on DelPhi in this work to demonstrate DelPhi’s capabilities in terms of accuracy, rate of convergence and speed of calculations. It was shown that DelPhi is a robust solver and capable of solving various biological applications. The benchmarks confirmed that DelPhi delivers energies that are almost grid independent, reaches convergence at scales equal to or larger than 1–2 grids/Å, and the speed of calculations is impressively fast. Finally, as shown in comparison with analytical solutions, the algorithm is, most importantly, capable of providing accurate energy calculations.

## Availability and requirements

Project name: DelPhi

Project home page: e.g. http://compbio.clemson.edu/delphi.php

Operating system(s): Linux, Mac, Windows

Programming language: Fortran and C

Other requirements: no

License: free of charge license is required

Any restrictions to use by non-academics: Commercial users should contact Accelrys Inc.

## Competing interests

The authors declare that they have no competing interests.

## Authors’ contributions

LL analyzed the data, drafted the manuscript and maintains DelPhi package. CL maintains the DelPhi package and helped writing the manuscript. SS, JZ, SW, ZZ, LW and MP developed and maintain DelPhi web server and website and associated resources. EA supervised DelPhi development and maintenance and finally draft the manuscript. All authors read and approved the final manuscript.
